# Anticancer Potential of Cherry Extracts and Major Flavonols: Focus on Quercetin and Kaempferol Molecular Mechanisms in Breast Cancer

**DOI:** 10.3390/ijms27187999

**Published:** 2026-09-08

**Authors:** Evangelia K. Konstantinou, Kallirroi Dimiza, Maria Dimitriou, Anastasios Darras, Athanasios A. Panagiotopoulos

**Affiliations:** 1 Department of Nutritional Science and Dietetics, School of Health Sciences, University of the Peloponnese, Antikalamos, 24100 Kalamata, Greece; e.konstantinou@go.uop.gr (E.K.K.); m.dimitriou@go.uop.gr (M.D.); 2 Department of Agriculture, School of Agriculture and Food, University of the Peloponnese, Antikalamos, 24100 Kalamata, Greece; k.dimiza@uop.gr (K.D.); a.darras@uop.gr (A.D.)

**Keywords:** breast cancer, cherries, phytochemicals, polyphenols, flavonols, quercetin, kaempferol, chemoprevention, apoptosis, cytotoxicity

## Abstract

Breast cancer remains one of the leading causes of global mortality, which has growing driving interest in dietary natural products for potential chemopreventive and complementary approaches. Cherries (*Prunus* species) are rich in bioactive phytochemicals, notably anthocyanins, hydroxycinnamic acids, and the flavonols quercetin and kaempferol. This review aims to examine and discuss current research on the molecular targets and mechanisms of action of whole cherry extracts and their primary flavonols quercetin and kaempferol across various breast cancer subtypes. Preclinical studies indicate that these compounds exert multi-targeted effects by inducing apoptosis, inhibiting cell proliferation, and suppressing metastatic pathways. Additionally, these flavonols show potential in reversing multidrug resistance. A whole-food approach highlights the candidate synergistic properties of these phytochemical complexes, but low oral bioavailability and rapid metabolism severely limit their clinical translation. Therefore, utilizing innovative drug delivery systems remains crucial to improving their stability, absorption, and overall therapeutic efficacy.

## 1. Introduction

Breast cancer (BC) is one of the most common causes of death across the globe [1,2,3,4,5,6,7,8,9,10,11,12,13]. Notably, in the last few years, many novel chemopreventive strategies have been developed that are based on using dietary natural products in the management of breast cancer [1,2,14,15,16,17,18,19,20,21,22,23,24]. In the case of bioactive polyphenolic compounds from cherries and their derivatives, several studies have been carried out on favorable biological outcomes [25,26,27,28]. Cherries contain phenolic compounds, including flavonoids, anthocyanins, and other bioactive molecules, which have been linked to favorable health effects [25,26,27,28]. Among various dietary sources, cherries (*Prunus* species) have emerged as an exceptional source of bioactive phytochemicals [29,30]. Accumulating evidence suggests that their regular consumption or the administration of their concentrated extracts can interfere with breast cancer development, representing a promising candidate approach for complementary research strategies with low cytotoxicity in non-cancerous cells [29,30].

In vitro cell lines have become an indispensable tool in breast cancer research for understanding the disease and its treatment, as well as for systematically evaluating the efficacy of these dietary compounds [31,32,33,34,35,36]. Such well-characterized human tumor populations are established cell models that can be grown under laboratory conditions, including serial replication, thus enabling researchers to explore specific molecular mechanisms, cytotoxicity, and intracellular signaling pathways [31,32,33,34,35,36]. These series provide an extremely useful and necessary resource for studying mechanisms associated with breast cancer [31,34,35]. Many cell lines used in the laboratory display characteristics that fall within the subtypes of human breast cancer found clinically [33,35,36]. It is, of course, through this connection with clinically recognized subtypes that researchers are able to study how different treatments affect different types of cancerous cells [33,35,36]. Therefore, comprehending the multi-factorial nature of the ailment relies on cell lines representing a variety of biological characteristics of the subtypes of cancer; the variances can be pronounced [35,37,38,39,40,41]. As these cell lines can be infinitely replicated under cultured conditions, they constitute a homogeneous population for experimentation and testing and turn out to be dependent and forever accessible sources for study [39]. This reproducibility is particularly crucial for analyzing new drugs and treatments since it allows a statistically reliable evaluation of their efficacy and reactions [35,39]. MCF-7 is the most commonly used breast cancer cell line in the entire world [31]. Owing to the highly specific sensitivity towards estrogen of MCF-7, it finds maximal application and makes an exemplary research tool in hormonally dependent breast tumors [31]. Outside of MCF-7, many other cell lines are being used to study different aspects of the disease [35,39]. MDA-MB-231 and MDA-MB-468 are more aggressive and are used in investigations related to metastasis and therapy resistance [42,43,44,45,46,47,48,49,50]. BT474 is a cell line representing the Hormone Receptor-positive (HR+)/Human Epidermal Growth Factor Receptor 2-positive (HER2+) subtype of luminal B, while MDA-MB-453 represents Hormone Receptor-negative (HR-)/HER2+ (HR-/HER2+, AR+ molecular apocrine breast cancer cell line), and MDA-MB-231 represents HR-/HER2-triple-negative breast cancer (TNBC) cell line [51,52,53]. These lines provide critical data among the aggressive series of cancer types, which can be important for the study of resistance and cell migration and invasion in vitro mechanisms [51,52,53]. The other cell line that can be used for comparison in this case is the MCF-10A cell line, which is a non-tumorigenic, immortalized human mammary epithelial cell line [54,55]. The action of cherry polyphenolic compounds on the above cell lines is discussed in the sections below.

Cherries are rich in phytochemicals, mainly anthocyanins, flavonols and hydroxycinnamic acids, which are associated with their bioactivity profile in preclinical models [56]. However, the qualitative and quantitative profile of these bioactive compounds is not uniform. It varies greatly depending on the particular cultivar (e.g., sweet cherry, *Prunus avium*, or sour cherry, *Prunus cerasus*) and environmental factors including geographical location, climate, soil fertility and stage of ripening at harvest [57]. These variations, therefore, have a direct impact on antioxidant capacity and, consequently, on the cytotoxic potency of cherry-derived extracts against breast cancer cells, a point to be taken into account when assessing pre-clinical data [29].

Among the polyphenols present in cherries (*Prunus* species), the flavonols quercetin and kaempferol are of particular importance. They are mainly present as glycosides such as quercetin-3-O-rutinoside, quercetin-3-O-glucoside and kaempferol-3-O-rutinoside, which are the major flavonols in sweet (*Prunus avium*) and sour (*Prunus cerasus*) cherries [29,30]. Preclinical research has largely centered on two representative flavonols for potential interactions with key breast cancer pathways, including hormone receptor signaling, survival cascades, and drug resistance mechanisms [56]. By focusing on quercetin and kaempferol, a useful framework is provided for exploring the bioactive potential of cherry phenolics.

This review aims to survey current research on the identified molecular targets and modes of action of whole cherry extracts, bioactive fractions, and their prominent flavonols, specifically quercetin and kaempferol, in the context of experimental breast cancer models.

## 2. Whole Cherry Extracts and Bioactive Fractions: Phytochemical Composition and Oncological Targets

### 2.1. Phytochemical Characterization and General Cytotoxic Mechanisms

Cherries are recognized as a rich source of bioactive phenolic compounds including flavonols, catechins, proanthocyanidins, anthocyanins, anthocyanidins and non-flavonoid phenolic compounds, and their metabolites [58,59,60,61,62]. Quantitatively, cherries are mostly rich in hydroxycinnamic acids, which represent the major class of phenolic acids in these fruits [58,59,60,61,62]. According to comprehensive phytochemical analyses, cherries exhibit two main hydroxycinnamates: neochlorogenic and *p*-coumaroylquinic acids [58,59,60,61,62]. Notably, quantities of melatonin, the neurohormone generated by the pineal gland and in charge of controlling a number of biological and physiological functions in the body, are also present in cherries [62]. These interrelated substances can modulate several important biological pathways related to breast cancer cell signaling, differentiation, cell cycle arrest, apoptosis, and metastasis in the context of oncology [63].

Reddy et al. [64] were the first to suggest that cherry pigments are multi-targeted. They studied the biological properties of natural food pigments including betanin, cyanidin-3-O-glucoside, lycopene, bixin, chlorophyll and *β*-carotene to assess their ability to inhibit the activity of lipid peroxidation, cyclooxygenase enzymes 1 and 2 (COX-1 and COX-2) and cancer cell growth [64]. These pigments were extracted from different plant matrices and used alone or in mixtures to assess their synergistic effect [64]. Cyanidin-3-O-glucoside is an anthocyanin pigment from *Prunus cerasus* (sour cherry) and a water-soluble pigment with strong antioxidant activity and inhibitory activity against inflammation [64]. Cyanidin-3-O-glucoside at 100 µg/mL showed a significant inhibitory effect on COX-2 (59%), while COX-1 inhibition was lower (29.5%) [64]. COX-2 is an enzyme related to inflammation and cancer; thus, this incubation demonstrates an anti-inflammatory response in vitro similar to classic cyclooxygenase inhibitors [64]. The COX-2 inhibitory action of cyanidin-3-O-glucoside in evaluations for cancer cells showed a strong effect against lung cancer cells, with a half-maximal inhibitory concentration (IC_50_) = 158 µg/mL [64]. In later confirmatory evaluations, the compound had a strong inhibitory effect against HCT-116 colon cancer cells (40%) and MCF-7 breast cancer cells (25%) [64].

In addition to whole-fruit dynamics, Ogur et al. [65] explored the effects of cherry consumption on certain multi-drug-resistant bacteria, asymmetric di-methylarginine (ADMA) levels and breast cancer cell lines. After treatment with cherry fruit extracts, the cancer cell proliferation activity was measured and the apoptotic cell nuclei were counted by using the 3-[4,5-dimethylthiazol-2-yl]-2,5-diphenyltetrazolium bromide (MTT) assay [65]. Minimum inhibitory concentration was determined by the microdilution broth method and ADMA levels were determined by high-performance liquid chromatography (HPLC) [65]. In vitro, cherry fruit extracts exhibited anti-bacterial activity against multi-drug-resistant bacteria and anti-proliferative activity against mouse mammary tumor cells (4T1) and human breast mammary adenocarcinoma cells (MCF-7) [65]. Cherry extract also induced apoptosis and decreased ADMA concentrations in treated cultures [65]. Ogur et al. concluded that the results of the study may contribute to the studies on the use of dietary supplements of traditional anti-inflammatory foods as potential preventive dietary components warranting further investigation in cardiovascular, oncological, and antibacterial research [65].

Maragheh et al. [66] studied the anticancer effects of Sour Cherry Pit Oil (SCPO) in breast cancer. The stable SCPO nanoemulsion (SCPO-NE), with the ultrasmall particle size of 36.5 nm and high stability, was prepared by the ultrasonic method [66]. The cytotoxic activity of SCPO-NE was tested in breast cancer cell lines (MCF-7) and in breast cancer mouse model (inoculated with TUBO-Mouse mammary tumor cancer cells) [66]. SCPO-NE significantly reduced the proliferation of cancer cell lines and decreased their viability by 50% compared with human foreskin fibroblasts (HFF) [66]. This reduction was due to the apoptosis mechanism, in which SCPO-NE activated specific pathways to induce cell death [66]. When administered to the experimental mouse model, SCPO-NE led to a smaller tumor, showing its anti-tumor effect in in vivo models [66]. CPO-NE induced apoptosis (programmed cell death) by activating caspases (e.g., caspase-33) [66] and modulating the balance of pro- and anti-apoptotic proteins. Such properties of SCPO-NE in targeting malignant cells without harming healthy cells have been further demonstrated by research and demonstrate promising cytotoxic activity in preclinical models [66]. Based on these results, SCPO-NE represents a novel candidate formulation for targeted antitumor research, displaying selective cytotoxicity in vitro and in vivo [66]. The study has demonstrated positive effects and has encouraged additional research on the long-term safety and effectiveness of SCPO-NE in models for various cancers [66].

To circumvent the gastrointestinal degradation of these fragile phenolics, Ždero Pavlović et al. [67] extracted phenolic compounds from sour cherry (*Prunus cerasus* L.) waste and tested their potential as bioactive compounds with anticancer activity. The compounds were isolated and later microencapsulated with maltodextrin by using freeze-drying (lyophilization), and the stability of the microencapsulated compounds during in vitro digestion was examined [67]. The results suggested that microencapsulation may provide significant levels of protection to bioactive compounds during digestion [67]. The antiproliferative and cytotoxic effects of the microencapsulated compounds and extracts were tested in cancer cell lines HT-29 (colon cancer) and MCF-7 (breast cancer) [67]. The results suggested that the encapsulation of polyphenols can improve their stability and antioxidant and anticancer activity, indicating that sour cherry compounds may be a potential source for the development of functional foods [67]. The anticancer effects of phenolic compounds isolated from sour cherry residues are related to their potent antioxidant potential and their ability to modulate specific cellular proteins that regulate the proliferation of cancer cells [67]. Phenolic compounds, such as anthocyanins and caffeic acid derivatives, mainly act by inhibiting oxidative stress and DNA damage through the scavenging of free radicals [67].

In particular, composition changes the expression and activity of important proteins such as p53, known as the “guardian of the genome” and responsible for DNA repair and apoptosis, as well as proteins that regulate apoptosis such as B-cell Lymphoma 2 (Bcl-2) and Bcl-2 Associated X-protein (Bax) [67]. The reported observations have shown that sour cherry phenolic compounds can increase the expression of the pro-apoptotic protein Bax and reduce the expression of the anti-apoptotic protein Bcl-2 to enhance apoptosis in cancer cells [67]. Similarly, the phenolic compounds derived from sour cherries can affect mitogen-activated protein kinases (MAPKs) and phosphatidylinositol 3-kinase/protein kinase B (PI3K/Akt) activity, signaling pathways that play an important role in cancer cell survival and proliferation [67]. Effectively, inhibition of MAPKs and PI3K/Akt contributes to reduced cell proliferation and increased apoptosis, resulting in the enhanced antitumor activity of the sour cherry extract [67]. Microencapsulation provides protection for polyphenolic compounds and increases stability and bioavailability of the compounds [67]. Subsequently, efficacy against cancer cells increases throughout the digestion and absorption in the body [67].

### 2.2. Molecular Pathways Targeted by Cherry Phenolics in Aggressive Breast Cancer Subtypes

The therapeutic window of the cherry extracts is particularly evident when evaluating different clinical subtypes of breast cancer, from hormone-dependent models to aggressive phenotypes. Layosa et al. [68] researched the anticancer effects of *Prunus avium* (otherwise known as dark sweet cherry, DSC) phenolic compounds. In particular, this research was conducted on MDA-MB-453 breast cancer cells, which serve as a model for HER2-overexpressing malignancies [68]. Phenolic compounds of DSC, in particular anthocyanins, trigger the activation of MAPK pathways, suppress Akt and phospholipase C-gamma-1 (PLCγ-1) activity, and combine with apoptosis in malignant cells [68]. They also induce apoptosis by enhancing the ratio of Bax/Bcl-2 up to 2 and specifically cleave the protein PARP-1, indicating significant pro-apoptotic and antiproliferative effects in vitro [68]. DSC’s phenolic compounds have also been proven to diminish the invasive ability and in vitro migratory capacity of malignant cells [68]. Furthermore, anthocyanins exhibited a greater potency on sustained activation of the extra-cellular signal-regulated kinases 1/2 (ERK1/2) and p38 MAPK pathways, and inhibition of pathways further confirmed the apoptotic process associated with the ERK1/2-Akt and MAPK pathways [68]. The research findings suggest that DSC phenolic compounds represent candidate dietary bioactive agents against breast cancer, which is supported because they target various cellular pathways associated with apoptosis and carcinogenesis [68]. These findings confirm the general potency of DSC phenolic compounds, especially anthocyanins, with potent anticancer activity on MDA-MB-453 breast cancer cells [68]. These bioactives could act on multiple molecular pathways to induce apoptosis and inhibit the invasive potential of MDA-MB-453 cancer cells [68].

Validating these findings in an in vivo setting, Noratto et al. [69] investigated the antitumor properties of phenolic compounds in DSC using experimental animals inoculated with human breast cancer cells (MDA-MB-453). The study demonstrated that the administration of total phenols, anthocyanins, and proanthocyanidin extracts to mice for 36 days suppressed the growth of tumors without toxic side effects [69]. In detail, the extracts induced activation of the apoptosis-related ERK1/2 pathway, while anthocyanins further exerted their inhibiting action against proteins associated with tumorigenesis and inflammatory responses [69]. The histological study demonstrated that extracts inhibited the proliferation of cancer cells, with anthocyanins being the most active [69]. Quantitative proteomic analysis revealed that 66 poor prognosis-related proteins of breast cancer were only expressed in the control group, which suggests that anthocyanins from cherries targeted proteins most involved in mechanisms of tumor growth and dissemination [69]. These data establish anthocyanin extracts from cherry as potential leads for further preclinical evaluation in breast cancer and encourage further investigations to confirm anti-invasive activity in more aggressive models of the disease [69].

To address the most recalcitrant subtype, triple-negative breast cancer (TNBC), Silveira Rabelo et al. [70] examined the anticancer activity of anthocyanins from the DSC against advanced breast adenocarcinoma. In this study, both total polyphenols and anthocyanin-rich extract of DSC were tested using the 4T1 breast cancer cell line as a murine syngeneic model of TNBC [70]. In the case of this study, anthocyanins were more effective in reducing cell viability compared to total polyphenols [70]. The IC_50_ value was recorded to be 58.6 µg/mL for the anthocyanin [70]. Anthocyanins from dark cherries demonstrated higher potency in inhibiting the viability of 4T1 cancer cells compared to total polyphenols [70]. Indeed, these also worked to reduce tumor levels of reactive oxygen species (ROS) responsible for cellular growth and survival of cancer cells through the activation of pro-growth signaling pathways [70]. Moreover, the anthocyanins affected the apoptosis pathway by acting on caspase-3 activation and the total PARP-1 protein reduction [70]. Caspase-3 is an apoptotic executioner, and since the PARP-1 is cleaved, DNA repair systems were inactivated and that consequently led to cell death [70]. The study also showed that anthocyanins did not stimulate caspase-8 activity, which is the partner of the extrinsic (outside the cell) apoptotic pathway, but the method of activation that is relevant at this time is the intrinsic or mitochondria apoptotic pathway [70]. The pathway of ERK1/2 is important to induce cell infiltration and decrease cell death [70]. The percentage of the phosphorylated form of ERK1/2 (p-ERK1/2) was decreased in the case of anthocyanins, which synchronizes the activities of this pathway [70]. Therefore, reducing the phosphorylated form of CREB (cAMP Response Element-Binding protein) transcription factor appeared to be enough to initiate gene expressions related to cell division and survival [70]. The protein kinase B/mechanistic target of rapamycin (Akt/mTOR) is the second most critical signaling pathway that enhances cell growth, survival and metabolism [70]. In TNBC, the Akt/mTOR pathway is usually hyperactivated and contributes to tumorigenesis and drug resistance [70]. Anthocyanins suppressed the Akt/mTOR pathway and reduced phosphorylation of Akt and mTOR proteins and ribosomal protein S6 (RPS6) [70]. Anthocyanins are attributed to the dual inhibition of ERK1/2 and Akt/mTOR pathways, which halts the activation of compensatory routes and augments antitumor activity [70]. Anthocyanins also decreased mRNA expression of vascular cell adhesion molecule-1 (VCAM-1), a cell adhesion molecule suspected to play a role in cell adhesion and invasive potential [70]. They also downregulated the phosphorylation of Src tyrosine kinase (Src), a protein enzyme associated with the activation of the Akt pathway, which further promotes cell transformation through epithelial-to-mesenchymal transition (EMT) and increases motility and invasiveness of cancer cells [70]. This study suggested that anthocyanins from DSC could act as effective inhibitors of triple-negative breast cancer cell lines in vitro by inhibiting central signaling pathways and promoting cancer cell apoptosis [70].

### 2.3. Management of Treatment-Related Adverse Effects and Supportive Care

In parallel to tumor reduction in pre-clinical models, the management of the adverse effects caused by clinical oncological treatments is a parallel therapeutic approach. Shenouda et al. [71] examined the effects of tart cherry concentrate on aromatase inhibitor-induced arthralgia (AIA) in patients diagnosed with hormone-positive breast cancer. AIA resulting from the use of aromatase inhibitors is a common adverse event that can result in unplanned discontinuation of aromatase inhibitor therapy, leading to decreased survival of patients [71]. The active mechanism of action of cherries is largely due to their high content of bioactive phytochemical compounds (anthocyanins, hydroxyl-cinnamates, and flavan-3-ols) that characterize the potential anti-inflammatory effects of tart cherries and may alleviate pain due to estrogen deficiency, an important mechanism contributing to AIA development [71]. Cherries have been shown to be associated with decreased secretion of pro-inflammatory cytokines, including tumor necrosis factor-alpha (TNF-α) and interleukin-1 beta (IL-1β), produced in several low-grade inflammatory processes [71]. The anti-inflammatory effects of cherries were also attributed to anthocyanins, which have been compared to non-steroidal anti-inflammatory drugs (NSAIDs) such as ibuprofen and naproxen for their comparable bioactivity via inhibition of COX-1 and COX-2 enzymes [71]. In the study, patients who consumed the available cherry concentrate showed marked pain improvement when compared to the placebo; however, no significant changes in C-reactive protein (CRP) levels, a marker of systemic inflammation, were observed [71]. This finding implies pain relief is not necessarily linked to the systemic anti-inflammatory action of bioactive compounds found in cherries but rather may be attributable to other properties of the bioactive compounds or local mechanisms [71].

## 3. Major Bioactive Flavonols in Cherries and Their Molecular Mechanisms

A subclass of the flavonoids group, flavonols share molecular similarities with flavones but differ in having an additional hydroxyl at position 3 of the C ring (3-hydroxyflavone), the primary backbone of the flavone [72]. The most prevalent flavonols in all varieties of cherries are quercetin analogs, along with kaempferol analogs and rutin (a glycoside derivative of quercetin) [60,61,63,73,74,75]. Other related flavonoid subclasses present in cherries include flavanones (such as naringenin) and flavanonols/dihydroflavonols (such as taxifolin) [60,61,63,73,74,75]. Flavonols (aglycones and glycosides) can be found in cherries at concentrations of up to 520 mg/L [60,74,76], whereas quercetin can range from about 27 to 213 mg/L in cherries from different regions of the world [60,74,76]. Temperature and UV radiation are usually associated with flavonoid degradation, and flavonoid content appears to be correlated with the amount of sun exposure received by the fruit during cultivation [77,78]. Furthermore, the production of cherry juice and oils and their storage all significantly affect flavonol concentration [74].

The effects of the most abundant flavonols in cherries have been investigated for their potential chemopreventive and antiproliferative activities in preclinical models [79,80,81,82,83,84,85,86,87,88,89]. Although these compounds share a high structural similarity, their bioavailability as well as their mechanisms of action can vary depending on the metabolites produced and the individual substitutions present [79,80,81,82,83,84,85,86,87,88,89]. Anticancer activities of flavonols have been explored in various cancers such as breast, prostate, ovarian, liver, colon, and bladder cancers [90,91,92,93,94,95]. Specifically, many studies on breast cancer have been carried out since much emphasis has been placed on breast cancer due to the involvement of diet in this disease [96]. The range of results of the above studies varies regarding cancer prevention, with null or slightly positive results being reported [83,97,98]. It is worth noting that the use of flavonoids as anticancer agents in preclinical and clinical studies is limited, in contrast to their use in basic anticancer research [85,99].

There are three primary activities of flavonoid compounds in breast cancer: (a) induction of programmed cell death; (b) inhibition of cell growth; and (c) inhibition of the metastatic behavior of cancer cells [100,101,102,103,104,105,106,107]. Flavonoids activate mechanisms that facilitate the apoptosis of cancer cells and induce cancer cells to undergo apoptosis, ultimately limiting the spread of tumors [100,101,102,103,104,105,106,107]. Furthermore, flavonoids inhibit the growth of cancer cells by arresting the cell cycle and stopping cancer cell growth by preventing further reproductive cycles [100,101,102,103,104,105,106,107]. Finally, flavonoids inhibit the metastatic behavior of cancer cells, by decreasing the ability of cancer cells to invade other tissues and generate secondary tumors and subsequently perform the EMT, which is a critical step in the metastatic process [100,101,102,103,104,105,106,107].

In evaluating these preclinical data, it is important to note a clear methodological difference between studies using whole fruit extracts, phenol/anthocyanin-rich fractions, isolated individual flavonols (such as purified quercetin or kaempferol), and advanced formulations (such as nanoemulsions or microencapsulated extracts). Biological responses observed with purified compounds at high doses in vitro cannot be automatically attributed to whole cherries or crude extracts. In whole fruit matrices, individual flavonols occur at lower relative levels where a complex array of co-existing phytochemicals determines distinct matrix-bound bioavailability profiles and potential multi-component synergistic interactions.

### 3.1. Molecular Targets of Quercetin

Quercetin has demonstrated anticancer properties in various cancer types, including breast, bladder, prostate, ovarian, liver, and colon cancers [83,97,98]. However, despite the large number of published studies, few translational or clinical studies have evaluated quercetin as a therapeutic agent due to its low bioavailability (around 20%, with only 1% found in free form in serum) [83,85,97,98,99,108,109,110]. Quercetin has been used in several drug delivery systems, including in combination with doxorubicin, to improve its effectiveness [111,112,113]. Generally, the anticancer activities of quercetin in breast cancer can be organized into three groups: induction of apoptosis, growth inhibition (cell cycle arrest), and inhibition of metastatic behavior (invasion, migration, and EMT) [105,106,107]. Quercetin reduces the growth of breast cancer cells, such as MCF-7 and MDA-MB-231, at micromolar concentrations, with MCF-7 cells being more sensitive to quercetin than MDA-MB-231 [101,102,103,104].

Quercetin induces apoptosis through several pathways, including caspase activation via the mitochondrial pathway, inhibition of the Akt signaling pathway, and cell cycle arrest at the growth 2/mitosis (G2/M) phase through MAPK/Cyclin B1/Cyclin-dependent kinase 1 (CDK1) modulation [104,114,115,116,117,118]. It also induces intrinsic apoptotic pathway, increasing Bax expression and inhibiting Bcl-2 [81]. Additionally, its methylated metabolite, isorhamnetin, exhibits independent biological activity; for instance, co-treatment of isorhamnetin with chloroquine was shown to induce mitochondrial fission via the Ca^2+^/calmodulin-dependent protein kinase II/Dynamin-related protein 1 (CaMKII/Drp1) axis, promoting apoptosis in TNBC cell lines [119]. Quercetin-3-O-d-galactopyranoside induced apoptosis via ROS by inhibiting the nuclear factor kappa light chain enhancer of activated B cells (NF-κΒ) signaling pathway and activating the Bax-caspase 3 axis [120]. Dietary quercetin has demonstrated an inverted “U”-shaped dose–response curve in the C3(1)/SV40 Tag breast cancer mouse model [121].

The antimetastatic potential of quercetin includes the inhibition of matrix metalloproteinase-3 (MMP-3) and matrix metalloproteinase-9 (MMP-9) expression, along with the inhibition of EMT and angiogenesis in breast cancer cells [114,115,122,123,124]. In MDA-MB-231 and MCF-7 cells, quercetin treatment reduced epidermal growth factor receptor (EGFR) levels, accompanied by distinct remodeling of membrane fatty acid profiles and alterations in free radical-driven lipid processes [125].

Quercetin has been reported as a multidrug resistance (MDR) inhibitor, acting as an inhibitor of p-glycoprotein through direct binding or downregulation of its expression [126,127,128]. It has also been shown to enhance the effect of doxorubicin and reduce its toxicity both in vitro and in vivo [110,129]. Similar effects were observed with other drugs, such as docetaxel, tamoxifen, paclitaxel, and vincristine, using various drug delivery systems [128,130,131]. The efflux transporters ATP-binding cassette subfamily G member 2 (ABCG2) and ATP-binding cassette subfamily C member 2 (ABCC2) mediate the cellular disposition and elimination of quercetin, kaempferol, and their conjugates, rather than participating directly in Phase II metabolism [132,133]. A functional interplay exists between Phase II conjugating enzymes, such as UDP-glucuronosyltransferases (UGTs), and ABC efflux transporters; UGTs generate glucuronidated metabolites, which are subsequently extruded from cells via ABCG2 and ABCC2, thereby limiting intracellular flavonol accumulation [132,133].

### 3.2. Molecular Targets of Kaempferol

Kaempferol has demonstrated anticancer properties in various cancer types, including breast, prostate, liver, and colon cancer, with particular emphasis on breast cancer [83,97,98]. However, due to its low bioavailability (around 2%), there are few preclinical and clinical studies using it as an anticancer agent [85,99,108]. The combination of kaempferol with other flavonoids, either in complex mixtures or simply with quercetin, has been reported to enhance its anticancer properties [134,135]. Kaempferol reduces the growth of breast cancer cells, such as MCF-7 and MDA-MB-231, at micromolar concentrations [105,106,107]. In a 3D MCF-7 model, extracellular signal-regulated kinase (ERK) signaling was responsible for apoptotic death and cell cycle arrest in the sub-growth 1 (sub-G1) phase [100,105].

Kaempferol inhibits the Ras homolog family member A (RhoA) and Ras-related C3 botulinum toxin substrate 1 (Rac1) signaling pathways, preventing cell migration and invasion in MDA-MB-231 and MDA-MB-453 cells [105,106,107]. It induces apoptosis via poly (ADP-ribose) polymerase 1 (PARP-1) cleavage, downregulates Bcl-2, and increases Bax [101,107]. Kaempferol has shown strong antioxidant activity, reduction in ROS-induced hemolysis and promotion of antiproliferative effects on cancer cell lines, including MCF-7 cells [136].

Molecular modelling and docking simulations indicate that kaempferol exhibits high binding affinity for estrogen receptor alpha (ERα) [137]. In functional assays, kaempferol displays concentration-dependent effects, functioning primarily through ERα-dependent mechanisms as an antagonist at lower concentrations (10 µM), whereas at higher concentrations (100 µM), it activates ER-independent alternative signaling pathways [138]. Kaempferol reverses the effects of xenoestrogenic compounds triclosan and bisphenol A, by increasing Bax levels and decreasing Bcl-2 levels, leading estrogen-responsive human breast cancer cell line VM7Luc4E2 to apoptosis [139,140]. It suppresses EMT and metastatic behaviors in MCF-7 cells induced by triclosan [141].

In a mouse xenograft model, kaempferol co-treatment inhibited tumor growth induced by 17β-estradiol (E2) or triclosan [139]. Kaempferol can reverse drug resistance promoted by the transporter ABCG2 [142,143], while inhibiting the efflux of quercetin by the same transporter [144]. The efflux transporters ABCG2 and ABCC2 participate in the in vivo disposition and excretion of kaempferol-3-glucuronide (the major metabolite of kaempferol) [132]. Cooperation between ABC transporters and UDP-glucuronosyltransferases regulates the cellular disposition of kaempferol conjugates, impacting their cellular accumulation [133].

The pharmacokinetic profile and physiologically achievable concentrations of quercetin and kaempferol need to be assessed to critically interpret these preclinical mechanisms. After oral ingestion both flavonols are extensively Phase II-conjugated in the intestine and liver producing mainly glucuronidated, sulfated and methylated derivatives, while circulating free aglycones remain at very low levels (<1–2%) [83,85,97,98,99,108,109,110]. Total flavonol metabolite post-prandial peak plasma concentrations (C_max_) after dietary or supplement intake are typically only low micromolar to nanomolar (0.1–1.5 μM) levels [83,85,97,98,99,108,109,110]. In contrast, the majority of in vitro breast cancer studies utilize unconjugated aglycones at concentrations ranging from 10 to 100 μM. This translational gap indicates that while high in vitro concentrations are crucial for elucidating cell-autonomous targets, their therapeutic relevance should be extrapolated cautiously unless verified with circulating metabolite profiles or in vivo systems.

**Figure 1:**
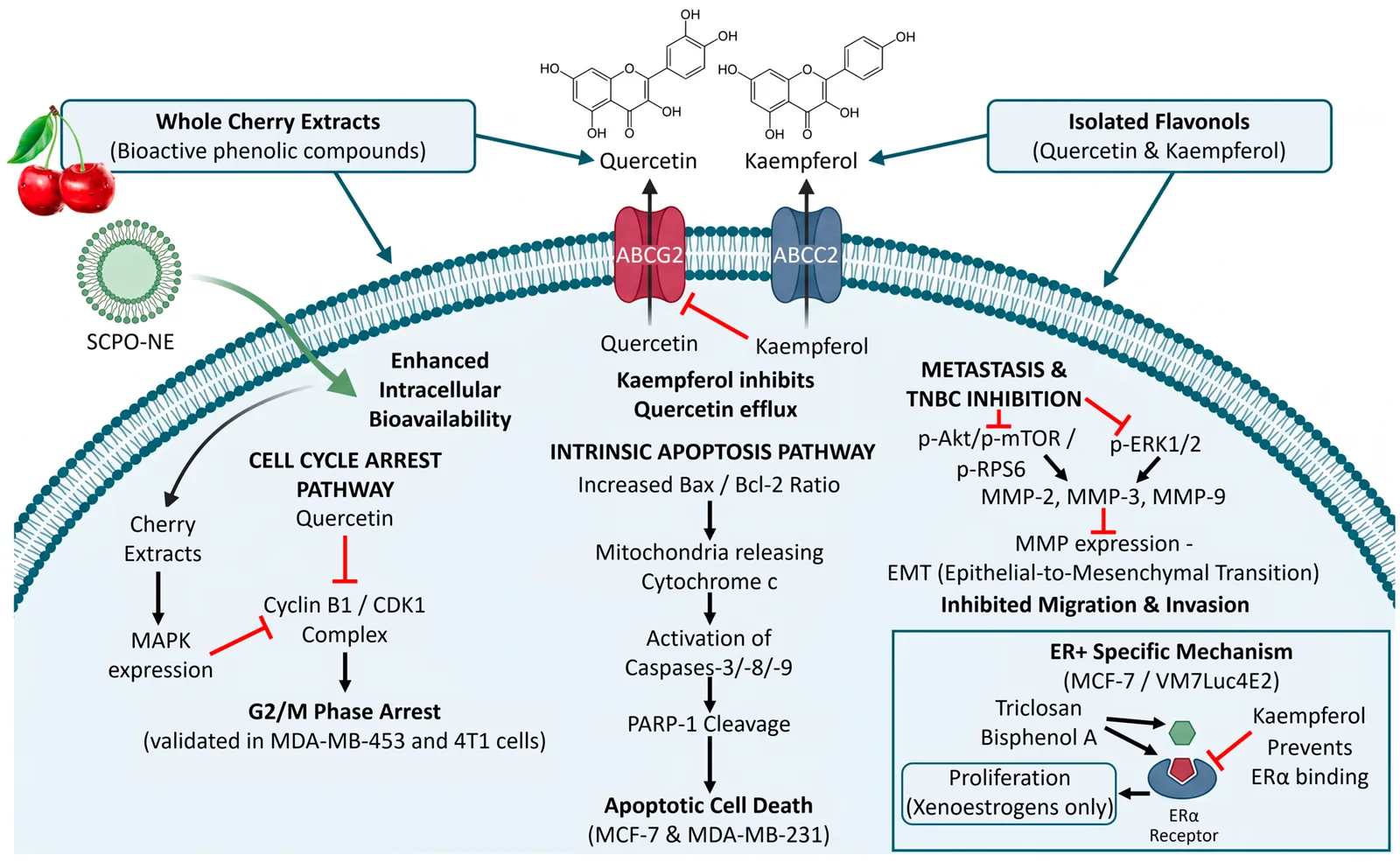
Integrative molecular mechanisms of whole cherry extracts and isolated flavonols (quercetin and kaempferol) in breast cancer. This figure integrates findings from diverse studies and cell lines; these pathways represent a broad spectrum of preclinical targets rather than a single, uniform mechanism. The schematic highlights: (Left) the enhancement of intracellular bioavailability via nanoformulations (SCPO-NE) and cherry extract-mediated G2/M phase arrest through MAPK/Cyclin B1/CDK1 modulation. (Center) intracellular drug retention dynamics, where kaempferol cooperatively blocks the ABCG2 transporter to inhibit quercetin efflux, leading to mitochondrial-mediated intrinsic apoptosis via Bax/Bcl-2 ratio upregulation, Cytochrome c release, Caspase-3/-8/-9 activation, and PARP-1 cleavage. (Right) attenuation of aggressive phenotypic markers associated with migration, invasion, matrix metalloproteinase (MMP-2/-3/-9) expression, and EMT via p-Akt/p-mTOR/p-RPS6 and p-ERK1/2 cascade inhibition, alongside the ERα-specific antagonistic mechanism of kaempferol against xenoestrogen-induced (Triclosan/Bisphenol A) proliferation.

Table 1 summarizes the experimental models, the doses administered, the key molecular targets, and the phenotypic effects of the action of various cherry extracts and pure flavonols, such as quercetin and kaempferol, on breast cancer. Figure 1 depicts the main intracellular signaling pathways through which cherry bioactive components induce apoptosis and inhibit the proliferation of breast cancer cells.

## 4. The Synergistic “Whole-Food” Approach and Bioavailability Challenges

### 4.1. The Synergy of Phytochemical Complexes

The antioxidant and antiproliferative effects observed in cherry extracts often surpass the effects of isolated fractions due to the natural synergy of phytochemical complexes [145,146]. Whole cherry extracts exhibit potential multi-targeted activity compared to single-molecule therapies, as anthocyanins, flavonols and hydroxycinnamic acids act simultaneously on distinct signaling cascades, preventing compensatory tumor mechanisms [147,148]. Moreover, it has been shown that mixing flavonoids enhances their anticancer properties, indicating the value of combinatorial approaches in the therapeutic use of these compounds [135,148].

### 4.2. Pharmacokinetic Barriers and Bioavailability Challenges

Despite the promising multi-targeted efficacy, the translation of cherry phenolics into clinical settings is largely hindered by severe pharmacokinetic limitations. The low bioavailability of prominent cherry flavonols such as quercetin and kaempferol greatly limits their efficacy as systemic therapeutic agents [108,109,110,149]. Kaempferol, in particular, has an oral bioavailability of only ~2% [150], whereas quercetin exhibits a baseline absorption of ~20% (depending on its sugar moiety) with the ingested dose undergoing rapid, extensive metabolism, leading to virtually no free aglycone in human serum [151].

These low systemic concentrations are primarily driven by extensive first-pass metabolism and active efflux systems. The ATP-binding cassette transporters, specifically ABCG2 and ABCC2, participate heavily in the in vivo transporter-mediated disposition and elimination of quercetin, kaempferol, and major metabolites such as kaempferol-3-glucuronide [152,153,154]. Furthermore, a strict intracellular cooperation between these ABC efflux pumps and phase II UDP-glucuronosyltransferases seems to tightly regulate the cellular disposition and excretion of these flavonol conjugates, thereby drastically limiting their cellular accumulation and therapeutic duration in malignant tissues [152,153,154].

### 4.3. Advanced Drug Delivery Systems to Overcome Clinical Limitations

To avoid these structural and metabolic barriers, recent oncological research has shifted towards innovative drug delivery systems engineered to safeguard fragile phenolics from gastrointestinal degradation, minimize efflux pump recognition, and maximize cellular uptake [155,156,157]. Advanced nanotechnology and structural encapsulation adopt necessary strategies to maximize the biological activity of cherry-derived components [158].

Promising strategies include loading quercetin and co-delivered anticancer drugs into targeted nanocarriers, which reduces the toxicity of chemotherapeutics and increases the tumor cytotoxicity [111]. In the case of cherry matrices, the microencapsulation of polyphenols by lyophilization with maltodextrin successfully protects sensitive polyphenols during in vitro digestion, increasing their downstream stability and antioxidant power [67]. Concurrently, the development of ultra-small and highly stable nanoemulsions such as the sour cherry pit oil nanoemulsion (SCPO-NE) has demonstrated promising efficacy in targeting breast malignant cells in experimental models, by modifying the balance of pro- and anti-apoptotic proteins, reducing tumor volumes while exhibiting selective cytotoxicity toward malignant tissues [66].

## 5. Conclusions

In conclusion, preclinical evidence suggests that whole cherry extracts and their primary flavonols, quercetin and kaempferol, exert their effects through multiple molecular pathways to inhibit proliferation of breast cancer cells, induce intrinsic apoptosis and decrease metastatic potential in diverse clinical subtypes, as well as to manage treatment-related side effects. The natural synergy of the cherry phytocomplex provides a promising multi-targeted bioactivity profile, but its therapeutic translation is severely restricted by low oral bioavailability, rapid phase II metabolism, and active transporter-mediated elimination by ABCG2 and ABCC2. Recent advances in drug delivery systems, specifically maltodextrin-based microencapsulation and stable sour cherry pit oil nanoemulsions (SCPO-NEs), successfully bypass these metabolic and transport barriers. These engineered platforms protect fragile phenolics from gastrointestinal degradation and maximize cellular accumulation inside malignant tissues, providing a promising framework for candidate complementary strategies with low toxicity in normal tissues.

## Figures and Tables

**Table 1:** Molecular targets, experimental models, and phenotypic outcomes of cherry extracts and major flavonols in breast cancer. ↓: Decrease; ↑: Increase.

Compound/Extract Form	Experimental Model & Cancer Phenotype	Dose/Concentration/Administration	Modulated Signaling Pathways & Molecular Targets	Phenotypic Outcome & Cell Fate	Ref.
**I. WHOLE EXTRACTS & COMPLEX BIOACTIVE FRACTIONS**					
**Cyanidin-3-O-glucoside** (from *Prunus cerasus*)	• MCF-7 (ER+)	• In vitro assays • 100 μg/mL	**Inflammatory Pathways:** ↓ COX-2 activity (by 59%) ↓ COX-1 activity (by 29.5%)	• Cyclooxygenase inhibition • Selective suppression of cancer cell growth	[64]
**Cherry Fruit Extracts**	• MCF-7 (ER+) • 4T1 (Murine mammary carcinoma/syngeneic mouse model)	In vitro assays	**Metabolic/Epigenetic Marks:** ↓ ADMA levels	• Anti-proliferative effect • Activation of apoptotic pathway	[65]
**Sour Cherry Pit Oil Nanoemulsion (SCPO-NE)**	• MCF-7 (ER+) • TUBO-expressing mice	• In vitro: Variable doses • In vivo: Oral/systemic	**Apoptotic Cascades:** ↑ Caspase-3 enzymatic cleavage ↓ Bcl-2/Bax expression ratio	• Modulation of mitochondrial survival markers • In vivo: Tumor growth inhibition and volumetric reduction in mouse models	[66]
**Microencapsulated Sour Cherry Phenolics** (Maltodextrin matrix)	• MCF-7 (ER+)	In vitro assays	**Survival Cascades:** ↑ Bax ↓ Bcl-2 ↓ MAPK ↓ PI3K/Akt phosphorylation	• Enhanced free radical scavenging • Intrinsic apoptosis induction • Growth arrest	[67]
**Dark Sweet Cherry (DSC)** (*Prunus avium*) (Phenolic/Anthocyanin Fraction)	• MDA-MB-453 (HR-/HER2+, AR+ molecular apocrine breast cancer cell line)	In vitro assays	**Stress & Survival MAPKs:** ↑ p38 MAPK ↑ ERK1/2 ↓ Total Akt & p-Akt ↓ Phospho-PLCγ-1 ↑ Cleaved PARP-1 ↑ Bax/Bcl-2 expression ratio (up to 2)	• Mitochondrial-mediated apoptosis • Attenuation of cell survival signaling • Inhibition of cell invasive capacity in vitro	[68]
**Compound/Extract Form**	**Experimental Model & Cancer Phenotype**	**Dose/Concentration/Administration**	**Modulated Signaling Pathways & Molecular Targets**	**Phenotypic Outcome & Cell Fate**	**Ref.**
**DSC Total Phenols, Anthocyanins & Proanthocyanidins**	• MDA-MB-453 tumor-bearing mice	In vivo Xenograft model	**Proteomic Landscape:** ↑ ERK1/2 signaling cascade ↓ Expression of 66 poor-prognosis oncogenic proteins	• Statistically significant tumor growth suppression in preclinical models • No observed treatment-induced systemic toxicity in animal models	[69]
**DSC Anthocyanin-rich Extract**	• 4T1 (Murine mammary carcinoma/syngeneic mouse model)	In vitro assays	**Dual Kinase Inhibition:** ↓ p-Akt ↓ p-mTOR ↓ p-RPS6 ↓ p-ERK1/2 ↓ p-CREB ↑ Caspase-3 & Cleaved PARP-1 ↓ ROS ↓ VCAM-1 ↓ p-Src	• Concomitant suppression of Akt/mTOR and ERK1/2 signaling pathways • Intrinsic apoptosis induction • Anti-metastatic & anti-inflammatory actions	[70]
**Tart Cherry Concentrate**	• Patients with Hormone-Positive (ER+) Breast Cancer	Clinical Context/Oral administration	**Systemic Inflammation & Cytokine Cascades:** ↓ Secretion of pro-inflammatory cytokines (TNF-α, IL-1β) ↓ COX-1 & COX-2 enzymatic pathways (NSAID-like bioactivity) - No significant modulation of systemic C-reactive protein (CRP) levels.	• Clinical management of Aromatase Inhibitor-Induced Arthralgia (AIA) • Marked pain improvement via localized anti-inflammatory mechanisms or estrogen-deficiency mitigation, independent of systemic inflammatory status	[71]
**Compound/Extract Form**	**Experimental Model & Cancer Phenotype**	**Dose/Concentration/Administration**	**Modulated Signaling Pathways & Molecular Targets**	**Phenotypic Outcome & Cell Fate**	**Ref.**
**II. PURIFIED FLAVONOLS: QUERCETIN & METABOLITES**					
**Quercetin** (Free Form)	• MCF-7 (ER+) • MDA-MB-231 (HR-/HER2- TNBC)	Micromolar (μΜ) concentration range	**Multi-Pathway Targeting:** ↑ Caspases-3, -8, and -9 ↓ Phospho-Akt (p-Akt) ↑ Bax/↓ Bcl-2 expression ↓ EGFR expression ↓ MMP-3 & MMP-9 secretion ↓ P-glycoprotein (P-gp/ABCB1)	• **MCF-7:** G2/M cell cycle arrest; Mitochondrial apoptosis • **MDA-MB-231:** Induction of intrinsic apoptotic pathway; Suppression of cell migration and invasion in vitro • **Chemoresistance:** Potential sensitization and reversal of Multidrug Resistance (MDR) in vitro	[81,101,102,103,104,114,115,116,117,122,123,124,125,126,127,128]
**Isorhamnetin** (Quercetin Metabolite)	• TNBC cell lines	Co-treatment with chloroquine	**Mitochondrial Dynamics:** ↑ CaMKII activation ↑ Drp1 phosphorylation ↓ Expression and activity of MMP-2 & MMP-9	• Induction of mitochondrial fission • Acceleration of the apoptotic cascade	[119,123]
**Quercetin-3-O-d-galactopyranoside**	• Breast Cancer Models	In vitro assays	**Transcription Factors:** ↓ NF-κΒ signaling pathway ↑ Bax/Caspase-3 axis	• ROS-mediated oxidative stress • Apoptotic cell death	[120]
**Dietary Quercetin**	• C3(1)/SV40 Tag transgenic mice	Ad libitum via diet (In vivo)	**Hormonal/Growth Tuning:** • Biphasic molecular modulation	• Exhibits a classic inverted “U”-shaped dose–response curve for tumor modulation	[121]
**Compound/Extract Form**	**Experimental Model & Cancer Phenotype**	**Dose/Concentration/Administration**	**Modulated Signaling Pathways & Molecular Targets**	**Phenotypic Outcome & Cell Fate**	**Ref.**
**III. PURIFIED FLAVONOLS: KAEMPFEROL & CO-TREATMENTS**					
**Kaempferol** (Free Form)	• MCF-7 (ER+) • MDA-MB-231 (HR-/HER2- TNBC) • MDA-MB-453 (HR-/HER2+, AR+ molecular apocrine breast cancer cell line) • VM7Luc4E2	Micromolar (μΜ) range (10 μΜ threshold)	**Cytoskeletal & Estrogenic Axes:** ↑ ERK phosphorylation (in 3D) ↓ RhoA & Rac1 GTPases ↑ Bax/Bcl-2 & PARP-1 cleavage ↓ ERα transcriptional activation ↓ ABCG2 (BCRP) pump	• **3D MCF-7:** Sub-G1 cell cycle arrest & ERK-mediated death • **Anti-metastasis:** Significant inhibition of breast cancer cell migration and invasion in vitro • **Endocrine Disruptors:** Attenuation of xenoestrogen (triclosan/BPA)-induced EMT & cell proliferation • **Chemoresistance:** Reversal of ABCG2-mediated resistance	[83,85,97,98,99,100,101,105,106,107,108,134,135,136,137,138,139,140,141,142,143]
**Kaempferol** **Co-treatment**	• MCF-7 (ER+) • In Vivo Xenograft Mouse Model (Estrogen-Responsive ER+)	Systemic Administration (Co-treatment via Injections/Diet)	**Inhibition of Estrogenic Signaling:** ↓ 17β-estradiol (E2) signaling ↓ Triclosan-induced proliferation	• Marked in vitro and in vivo inhibition of estrogen- or xenoestrogen-stimulated mammary tumor growth	[139]

## Data Availability

No new data were created or analyzed in this study. Data sharing is not applicable to this article.
